# Depression and parental distress among caregivers of autistic children: a serial mediator analysis in caregivers of autistic children

**DOI:** 10.1186/s40359-024-01704-x

**Published:** 2024-06-10

**Authors:** Dilara Demirpençe Seçinti, Dilan Diş, Zeynep Seda Albayrak, Ezgi Şen

**Affiliations:** 1 Şişli Etfal Research and Training Hospital, Child and Adolescent Mental Health, Istanbul, Turkey; 2 Department of Clinical Psychology, Istanbul Rumeli University, Istanbul, Turkey; 3 Department of Child and Adolescent Psychiatry, Koc University Hospital, Istanbul, Turkey

**Keywords:** Parental distress, Depression, Stigmatization, Autistic children

## Abstract

**Background:**

This study aimed to investigate the relationship between the severity of autism, emotional and behavioral problems of autistic children, internalized stigma, depressive symptoms, and primary caregiver parental stress. Specifically, we explored the mediating role of internal stigmatization and total difficulties of individuals with autism on parenting stress and depressive symptoms of the primary caregiver.

**Method:**

Mothers of 93 children with autism were included in the study. The mothers were given the Beck Depression Inventory (BDI), The Internalized Stigma of Mental Illness Scale (ISMI), the Autism Behavior Checklist, the Parenting Stress Index-Short Form, Strength, and Difficulties Questionnaire -Parent Form (SDQ-P).

**Results:**

As a result of our study, the emotional and behavioral problems of the child and the internalized stigmatization felt by the parent played a mediator role in the relationship between the child’s autism severity and the parent’s stress and depressive symptoms.

**Conclusions:**

Our findings highlight that internalized stigmatization and behavioral characteristics of individuals with autism are among the most critical problems for their primary caregivers. These results have important implications for the development of interventions aimed at reducing the internalized stigma experienced by primary caregivers of individuals with autism and for improving their mental health outcomes.

## Introduction

Autism is a neurodevelopmental disorder characterized by impaired social reciprocity, deficits in communication skills, and rigid and repetitive behaviors [1, 2]. Being the caregiver of a child with a neurodevelopmental disorder causes many negative emotions, such as guilt, worthlessness, denial, and disappointment from the first moment of diagnosis. The most important sources of difficulties felt by the family of a child with autism were considered to be the persistence of the disorder, the lack of acceptance of the child’s behavior in terms of society and family, and insufficient professional support [3].

One of the most significant society-based factors affecting a caregiver’s mental health is societal stigmatization.Discrimination, stereotypes, and negative judgments targeting a subgroup are defined as stigma. Society’s stigmatizing the disorders and projecting it onto the other person gradually becomes a self- stigma internalized by the target person [4]. The extent to which society internalizes and projects these stigmatizing attitudes significantly impacts the mental well-being of caregivers. As the person internalizes this stigma directed by society, accompanying depressive symptoms [5], negative self-evaluation of the person increases [6], and well-being is negatively affected [7]. All these challenges lead to matching the resources that the person has psychically with the demands of the situation he/she is in, leading to the formation of parental stress [8]. A metaanalysis study showed that parental stress of parents of autistic children is higher than in families of healthy children and even in families of children with developmental delays [9]. Studies have shown that the parental stress burden felt by the parents of autistic children is related to self-stigma [10], the severity of the child’s autism, and the severity of the child’s emotional and behavioral problems [11]. Besides the inability to regulate the child’s atypical behaviors, thinking about how the outside world judges these behaviors increases maternal stress. A study showed that the depression rates of mothers of children with autism are higher than the general population [12]. Another study showed that the mother’s self-stigma was effective on the depressive symptoms of mothers of children with autism [13]. Autism disorder is often accompanied by internalization (anxiety, depression, mood disorder) and externalization problems(aggression, conduct disorder, ADHD) [14]. Inappropriate behaviors of children with autism, who mostly do not have physical defects (hand clapping, self-harming movements, putting things in their mouths, aggressive behaviors) can be seen as inappropriate in society [15]. Research has consistently shown that children with autism often present with a range of emotional and behavioral difficulties [16–19]. Research has demonstrated that as the severity of autism in children increases, so do the associated emotional and behavioral problems [20].

Parents have reported feeling blamed by others for their children’s behavioral and emotional problems [20]. Disruptive behaviors and emotional challenges associated with autism may also lead to heightened stigmatization [21]. Research has shown that disruptive behaviors, such as aggression and non-compliance, are perceived as more dangerous and elicit a greater desire for social distance compared to withdrawn behaviors [22]. Additionally, emotional challenges experienced by individuals with autism, such as emotional dysregulation and behavioral problems, have been linked to increased parenting stress and poor understanding and acceptance by the public [23–25]. It has been noted that mothers of children with autism are more likely to perceive themselves as being stigmatized in relation to the contradiction between the normal appearance of the child and the severity of emotional and conduct problems of their children [24]. Therefore, understanding and addressing background history of the stigmatization are crucial in providing effective support for parents of children with autism.

In light of these findings, it is posited that the stigma experienced by the public towards families of children with autism extends beyond the autistic symptoms to include accompanying behavioral and emotional problems. This societal stigmatization is thought to contribute to depressive and stress-related symptoms in caregivers. It is suggested that while the severity of autism in children is linked to their emotional and behavioral issues, these problems are intertwined with stigmatization, which, in turn, is believed to exacerbate stress and depression in caregivers. Consequently, our study aims to employ serial mediator analysis to assess the presence of causal relationships among these mediators.

Our research is guided by two primary hypotheses:

**Hypothesis 1**

The severity of autism in children leads to emotional and behavioral problems, which in turn cause societal stigmatization. This stigmatization is hypothesized to increase depressive symptoms in caregivers. Severity of autistic symptoms→ Emotional and behavioral problems→ Internalized stigmatization → Depressive symptoms.

**Hypothesis 2**

The severity of autism in children leads to emotional and behavioral problems, which in turn cause societal stigmatization. This stigmatization is hypothesized to increase stress levels in caregivers.

Severity of autistic symptoms→ Emotional and behavioral problems→ Internalized stigma → Parenting Stress.

## Methods

### Participants and procedure

Mothers of children with autism between the ages of 6–11 participated in the study. These children with autism were individuals who sought medical reports for their children at the hospital. The medical report is a mandatory document for families with autistic children to integrate them into school. In this context, it was anticipated that the socioeconomic and educational levels of the participating families reflected those of general families of children with autism. Additionally, among the fundamental characteristics of children seeking a health report, it was required that the diagnosis of autism be made by a child psychiatrist according to DSM-5 criteria.

Mothers of children who had received a new diagnosis within the last year were excluded from the study. Mothers of children with autism were informed in writing and verbally by child psychiatrists regarding the study. The mothers did not receive any compensation for participating in the study. The scales provided for the study were filled out in a hospital setting. Due to the lengthy duration required for completing the scales, 10 mothers declined to participate in the study.

The study was designed as an exploratory study, and data were collected between 2021 and 2022. This research received approval from the Ethics Committee of *** University (N: 2020/15) and was conducted in accordance with the Declaration of Helsinki. Ninety-three mothers of autistic children participated in the study, and the age of the children ranged from 8.22 ± 3.63. Detailed demographic information about the participants is presented in Table 1.

### Measures

Sociodemographic Data Form: In the Sociodemographic Data Form, the age, gender, age at which child was diagnosed with Autism, comorbid psychiatric diagnosis, psychiatric drug use, medical illness, age of the mother, educational status, and the total number of children the mother had were asked (Table 1).

**Table 1:** Demographic information of the participants

Variables Continuous Variables	Mean(SD)
Age of the child	8.22 (3.63)
Time since diagnosis	
Age of the mother Categorical Variables	4.66 (3.27) 37.07 (6.10) n(%)
Gender of the child	
Male	74, (79.6%)
Having medical diagnosis	24 (25.8%)
Psychiatric drug use	55 (59.1%)
Education level of the mother	
Primary school education	47 (51.1%)
High school education	26 (28.3%)
University graduation- MA-PHD	19 (20.7%)
Number of the sibling of the autistic child	2.17 (0.91%)

### Strength and difficulties questionnaire -parent form (SDQ-P)

The SDQ-P, developed by Goodman, consisting of 25 items, was developed to measure children’s emotional and behavioral problems and prosocial behaviors [26]. It consists of five subscales: emotional symptoms (i.e.; Often unhappy, down-hearted or tearful), conduct problems (i.e.; Often has temper tantrums or hot tempers), hyperactivity/inattention problems (i.e.; Restless, overactive, cannot stay still for long), peer relationship problems (i.e.; Rather solitary, tends to play alone), and prosocial problems(i.e.; Considerate of other people’s feelings). At the same time, the total difficulty score is found by adding the emotional symptom, conduct problems, hyperactivity (inattention), and peer relationship problems. Turkish Parent Forms of SDQ Cronbach’s alpha values range from 0.84 to 0.37 (Total Difficulty Score 0.84, emotional symptoms 0.73, conduct problems 0.65, inattention / hyperactivity 0.80, Peer relationship 0.37, prosocial problems 0.73) [27].

### Autism behavior checklist (ABC)

The ABC scale was developed to measure the severity and frequency of autism in children and adolescents [28]. It is a 57-item assessment tool consisting of five subscales: sensory (Poor visual discrimination during learning (gets stuck on a feature such as size, color or position)), relating (Often does not pay attention to social/environmental stimuli), body and object use (S/he spins around her/himself for a long time), language skills (Does not follow simple commands once told (e.g. sit, come here, stand up)), and social and self-care skills (Learns a simple task but quickly forgets it). The lowest score that can be obtained from the scale is 0, and the highest score is 159. Cronbach’s alpha value was found to be 0.92 [29]. When the reliability findings were examined for the Turkish version of the scale, the Cronbach alpha coefficient and two-half test reliability were found to be 0.92 [29].

### The internalized stigma of mental illness scale (ISMI)

The Internalized Stigma of Mental Illness Scale was developed by Ritsher et al. to determine the internalized stigma of individuals with mental illness and consists of 29 items in a four-point Likert type, based on self-report [30]. The scale consists of 5 subscales: alienation(i.e.: I feel out of place in the world because I have a mental illness), confirmation of stereotypes(i.e.; Mentally ill people tend to be violent), perceived discrimination (i.e.; People discriminate against me because I have a mental illness), social withdrawal (i.e.; I avoid getting close to people who don’t have a mental illness to avoid rejection), and resistance to stigma (i.e.; People with mental illness make important contributions to society). The total score is obtained by summing the five subscales, and a high score indicates that the individual’s internalized stigma is more severe. For example, in one study, those above 2.5 were categorized as moderate and severe, and those between 2 and 2.5 were classified as mild [31]. Ersoy et al. carried out a validity and reliability analysis of the inventory in Turkish in 2007. The inventory’s Cronbach’s α coefficient comes out to be 0.89 [32].

### Beck depression inventory

It is a self-reported Likert-type scale consisting of 21 questions(i.e.; I feel discouraged about the future) and scored between 0 and 3 points. A high score indicates a high degree of depression [33]. For example, a score of 0–9 indicates normal, 10–18 mild depression, 19–29 moderate depression, and 30–63 severe depression.In the Turkish version of the inventory the test-retest stability was also good (*r* = 0.94), and the internal consistency for the nonclinical and clinical groups was 0.90 and 0.89, respectively [34].

### Parenting stress index-short form (PSI-SH)

PSI-SH is a Likert-type scale consisting of 36 questions and three subscales, obtained by shortening the long form of the Parenting Stress Form by Abidin [35].. There are Parental Distress(i.e.; I often have the feeling that I cannot handle things very well ), Parent–Child Dysfunctional Interaction (i.e.; My child rarely does things for me that makes me feel good), and Difficult Child subscales (i.e.; My child seems to cry or fuss more often than most children).The PSI-SF subdimensions in the Turkish version with test-retest correlation values of 0.58 for parental stress (PS), 0.69 for parent-child dysfunctional interaction (PCDI), 0.60 for challenging child (DC), and 0.91 for the entire index were determined [36].

### Data analysis

All statistical analyzes were performed using IBM SPSS Statistics for Windows, Version 22.0.

PROCESS function V.2.16.1 in SPSS V.21 was used for the mediator analysis.

The missing values of ISMI-TR, BDI, ABC, PSI are imputed using a regression analysis procedure. The missing values of SDQ-P were replaced using the guidelines recommend by the SDQ developers.The kurtosis and skewness tests were applied to the variables, and since the variables were found to be within the range of -1.5 and + 1.5, they were considered to follow a normal distribution [37]. Accordingly, parametric tests were conducted.

Post-hoc power analysis was performed with G*Power Version 3.1.9.4 [38, 39] to test the sample adequacy and detect the size’s putative effects. Fixed multiple based on six predictive variables, including a medium (≥ 0.15 Cohen, 1988) effect size estimate (f 2), two continuous mediator variables (SDQ-P and ISMI-TR), primary predictor (ABC), and two control variables. The regression model omnibus equation (deviation of r2 from zero) was calculated. Power analyses, using an alpha coefficient of 0.05 and 93 participants, revealed a power level equal to 0.98.

As a first step, a Pearson Product Moment Correlation Test (PPMC) correlation analysis investigated the relationship between parenting stress, depression severity, autism severity, emotional and behavioral problems of the child, and internalized stigmatization score. The strength of correlation coefficients was categorized as weak (*r* = 0.1–0.3), moderate (*r* = 0.4–0.6), strong (*r* = 0.7–0.9), and perfect (*r* = 1.0) [40].

As a second step, The variables that were significantly associated with each other were put into a serial mediation analysis in Process (Model 6) [41]. In the serial mediator analysis, the total depression score was entered as the dependent variable, the severity of autism was entered as the independent variable, child gender, child age, and presence of psychiatric treatment were entered as the covariants, internalized stigma and total problems were entered as the mediator, unlike the first, the parental stress score was entered as the dependent variable in the second serial mediator analysis.

## Results

### Preliminary analysis-correlation between variables

In our study, it has been shown that there is a moderate and strong relationship between SDQ-P, ABC, ISMI-TR, BDI, and PSI-SF (Table-2).

**Table 2:** Mean and standard deviation values of observed variables and the relationship between the variables

Observed Variables	Mean	SD	1	2	3	4
1.SDQ-P	20.08	6.14	-	-		
2. ABC	81.00	32.18	0.61**	-		
3. ISMI-TR	65.60	14.73	0.52**	0.38**		
4. BDI	20.59	9.95	0.42**	0.31**	0.50**	
5. PSI-SH	105.96	29.68	0.66**	0.52**	0.66**	0.61**

Note: SDQ-P: Strength and Difficulties Questionnaire Parent Form; ABC: Autism Behavior Checklist; ISMI-TR: Internalized Stigma of Mental Illness Scale Turkish Version; BDI: Beck Depression Inventory; PSI-SH: Parenting Stress Index Short Form; SD: Standard Deviation. (Significant in all correlations *p < 0.05, **p < 0.01). Controlled for gender, psychiatric treatment

### Primary analysis

**Fig. 1:**
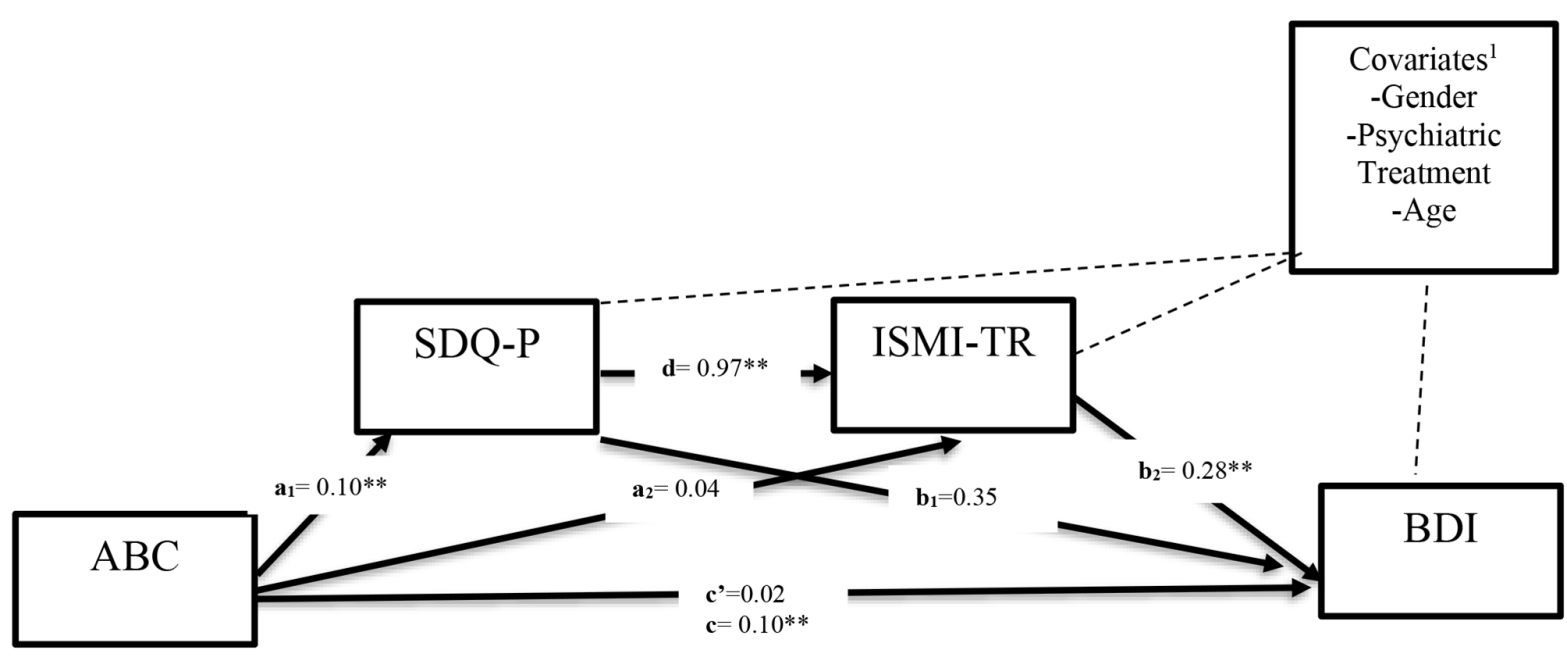
^1^Covariates are represented by dashed lines and were controlled for in all mediational pathways; SDQ: Strength and Difficulties Questionnaire; ABC: Autism Behavior Checklist; ISMI-TR: Internalized Stigma of Mental Illness Scale Turkish Version; BDI: Beck Depression Inventory. (Significant in all correlations *p<0.05, **p<0.01)

Two hypotheses were tested by serial mediator analysis. For each hypothesis, three models were tested, which are summarized below and depicted in Fig. 1 with a dotted line:

Hypothesis 1

Model 1: ABC(X)$\rightarrow$SDQ-P(M1)$\rightarrow$BDI (Y)

Model 2: ABC(X)$\rightarrow$ISMI-TR (M2)$\rightarrow$BDI (Y)

Model 3: ABC(X)$\rightarrow$SDQ-P(M1)$\rightarrow$ISMI-TR (M2)$\rightarrow$BDI(Y)

Hypothesis 2

Model 1: ABC(X)$\rightarrow$SDQ-P(M1)$\rightarrow$PSI (Y)

Model 2: ABC(X)$\rightarrow$ISMI-TR (M2)$\rightarrow$PSI (Y)

Model 3: ABC(X)$\rightarrow$SDQ-P (M1)$\rightarrow$ISMI-TR (M2)$\rightarrow$PSI(Y)

For the first hypothesis, Three models tested the indirect and direct effects between ABC and BDI scores; for the second hypothesis, three tested the indirect and direct effect between ABC and PSI scores. Finally, results are presented, which display the pathways and standardized coefficients.

### Result of hypothesis-1

Model 1: ABC scores as a predictor of SDQ-P scores and the three covariates were found to be significant F(4, 77) = 13.40, *p* ≤ 0.0001, R2 = 0.41. ABC scores significantly predicted SDQ-P scores(Path a1, b = 0.10 t(77) = 6.35, *p* ≤ 0.0001). In addition, gender (b = 0.50, t(77) = 0.37, *p* = 0.71) psychiatric treatment (b = 1.64, t(79) = 1.12, *p* = 0.27) and age (b = 0.29, t(77) = 1.93, *p* = 0.058) were insignificant covariates of SDQ-P scores.

Model 2: ABC scores as a predictor of ISMI-TR scores and the three covariates were found to be significant F(4, 77) = 5.88, *p* ≤ 0.0001, R2 = 0.27. ABC did not predict ISMI-TR scores(Path a2, b = 0.04 t(77) = 0.79, *p* = 0.43), and the association between SDQ-P and ISMI-TR scores (Path d, b = 0.97 t(77) = 3.13, *p* = 0.0024) was significant, F(5, 76) = 5.88, *p* ≤ 0.001. In addition, gender (b = -4.63 t(77) = -1.28, *p* = 0.21) psychiatric treatment (b = 2.38 t(77) = 0.76, *p* = 0.45) and age ( b = 0.26 t(77) = 0.63, *p* = 0.21) were not significant predictors of ISMI-TR scores (Fig. 1).

Model 3:ABC scores as a predictor of BDI scores and the three covariates were found to be significant F(6, 75) = 5.46, p ≤ 0.0001, R2 = 0.30.In the model testing the direct effect of ABC scores on BDI scores without any mediators included, ABC was not a significant predictor of BDI, ( Path c’,b = 0.023 t(77) = 0.63, *p* = 0.53 ). The total effect of ABC scores on BDI scores was significant (Path c, b = 0.10 t(77) = 2.96, *p* = 0.0041). ISMI-TR scores significantly predicted BDI scores (Path b2,b = 0.28 t(77) = 3.57, *p* = 0.0006 ). SDQ-P scores did not predict BDI scores (Path b1, b = 0.35 t(77) = 1.56, *p* = 0.12 ). There were no significant covariates in this model.

Indirect Effects. In our indirect effect models, SDQ-P scores did not significantly mediate the relationship between ABC scores and BDI scores with bootstrapped indirect effect point estimates B = 0.03, SE = 0.02, 95% bootstrapped CI [-0.008, 0.08]. Similarly, ISMI-TR scores did not significantly mediate the relationship between ABC and BDI scores, with a bootstrapped indirect effect point estimate = B 0.012, SE = 0.06, 95% CI [-0.02, 0.045]. However, it was found that both SDQ-P and ISMI-TR scores significantly mediated the relationship between ABC and BDI scores, yielding a bootstrapped indirect effect point estimate of 0.028, SE = 0.011, and a 95% CI of [0.008, 0.055].

**Fig. 2:**
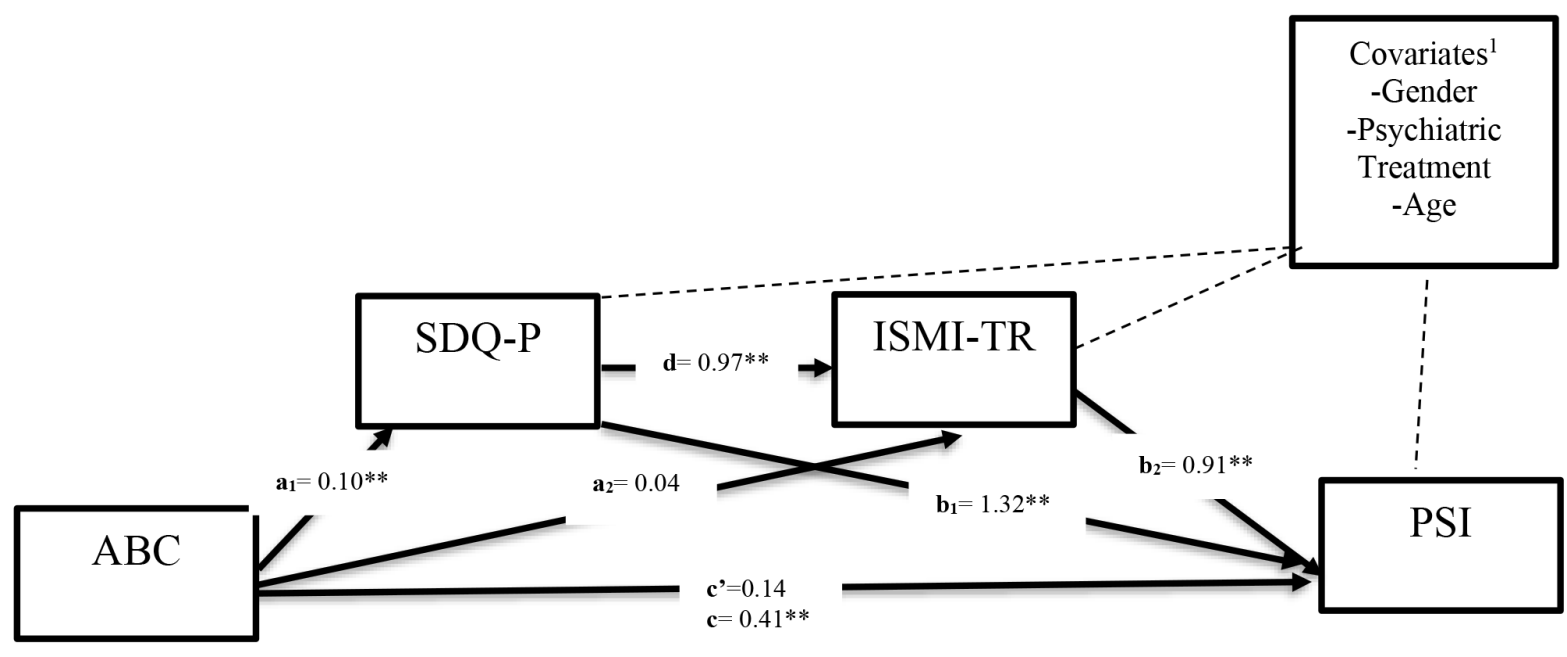
^1^Covariates are represented by dashed lines and were controlled for in all mediational pathways; SDQ: Strength and Difficulties Questionnaire; ABC: Autism Behavior Checklist; ISMI-TR: Internalized Stigma of Mental Illness Scale Turkish Version; PSI: Parenting Stress Index. (Significant in all correlations *p<0.05, **p<0.01)

ABC scores as a predictor of PSI scores and the three covariants were found to be significant F(6, 75) = 17.77, p ≤ 0.0001, R2 = 0.59. The direct effect of ABC scores on PSI scores was not significant when SDQ-P scores, ISMI-TR, and covariates were included in the equation(Path c’,b = 0.14 t(77) = 0.1.80, *p* = 0.074). The total effect of of ABC scores on PSI scores was significant (Path c, b = 0.41 t(77) = 4.98, *p* = 0.0001).ISMI-TR scores significantly predicted BDI scores (Path b2,b = 0.91 t(77) = 5.63, *p* = 0.0006 ). SDQ-P scores predicted PSI scores (Path b1, b = 1.32 t(77) = 2.84, *p* = 0.0057). There were no significant covariates in this model (Fig. 2).

Indirect Effects. In our indirect effect models, SDQ-P scores significantly mediate the relationship between ABC scores and PSI scores with bootstrapped indirect effect point estimates = 0.13, SE = 0.05, 95% bootstrapped CI [0.0417, 0.263]. Furthermore, ISMI-TR scores did not significantly mediate the relationship between ABC and BDI scores, with a bootstrapped indirect effect point estimate = 0.040, SE = 0.056, 95% CI [-0.0636, 0.160]. Lastly, SDQ-P and ISMI-TR scores significantly mediated the relationship between ABC and PSI scores, with a bootstrapped indirect effect point estimate = 0.092, SE = 0.034, 95% CI [0.032–0.167].

## Discussion

In the present study, we investigated the impact of the severity of autism in children through emotional and behavioral problems and internalized stigmatization on the formation of depressive symptoms and parental stresses of primary caregivers. In our study, it was shown that the severity of autism did not have a direct effect on the parental stress and depressive symptoms of caregivers. It was revealed that emotional and behavioral difficulties and internalized stigma of caregivers play a mediating role in the relationship between children’s autism severity and parental stress.

The initial finding of our study suggests that the severity of autism is not directly associated with depressive symptoms. However, it was demonstrated that autism severity leads to emotional and behavioral problems, subsequently contributing to depressive symptoms through the mechanism of stigmatization. In our study, we predicted that the severity of autism might increase depressive complaints, but we could not show a direct effect. A survey of the subject revealed that 70% of mothers had clinically significant depressive symptoms at the time of diagnosis of autistic children. Still, depressive symptoms continued at 30% after an average of 1.5 years [42]. In our study, the time of diagnosis of the patients coincided with an average of 4.5 years before the start of the study. For this reason, it was thought that the mothers in our study were accustomed to the autism core symptoms of their children and accepted them.Therefore, autism core symptoms did not directly affect the formation of depressive symptoms. However, studies have shown that the child’s behavioral problems [42, 43] and stigmatization play a role in the formation of the caregiver’s depressive symptoms in the period after diagnosis [40]. Therefore, in our study, it can be thought that mothers of children with emotional and behavioral problems with intense autistic symptom severity and mothers with more internalized stigmatization are at risk for depression. In other words, while stigmatizing the child’s core symptoms of autism does not pose a risk for depression, parents with stigmatization caused by emotional and behavioral problems are at risk for depression.

The second finding of our study indicates that, unlike the initial assumption, the severity of autism does not directly lead to parental stress. Instead, it induces parental stress through the intermediary of emotional and behavioral problems, culminating from the stigmatization associated with autism severity.Several studies show increased autism symptom severity will increase parental stress and cause mental health problems [44, 45].However, the fact that the sample of these studies is not from the general population but from hospital applications with a request for help is a limitation. Studies based on a population sample do not show a direct relationship between autism severity and parental stress [46, 47]. In addition, it has been shown that parental cognition can increase parental mental health problems by mediating between the child’s symptom severity and parental mental health problems [48]. Since the sample’s age range in the study was 6–11 years, it was thought that the result was compatible with the literature. Because in the articles examining the relationship between autistic symptom severity and parental stress, it has been shown that the direct relationship between autism core symptoms and parental stress is more robust in those with autistic children at a younger age, and behavioral and emotional problems play a role in the formation of parental stress with the increasing age of the child [49]. There are studies in the literature showing that emotional and behavioral problems of children with autism increase parental stress by increasing stigmatization. A study conducted in 2010 concluded that the increase in children’s behavioral problems increases the stigmatization that parents are exposed to in society, which in turn increases parental stress [50]. It has been stated that it increases parental stress by causing stigmatization of parents [51].

### Strengths and limitations

Our study is a cross-sectional study. Although the sample size of the current study is statistically sufficient, the more extensive sample set may enable parents to investigate which internalized stigma and emotional and behavioral problems are more challenging. In addition, the fact that the age groups of autistic children were in the 6–11 age range in the study sample caused the problems experienced by the families of autistic children during adolescence and young childhood not to be included in the article. Therefore, examining these age periods in future studies will enable us to understand the problems of families of autistic children according to the age groups of autistic children.

### Implications

Studies on stigmatization in families of children with autism have generally been conducted in Western cultures, and analyses in Eastern cultures are more limited [52]. For this reason, studies conducted in countries such as Turkey are valuable.

The findings of our study showed that the child’s problematic behaviors were highly effective on caregiver depression and parental stress by causing internalized stigma. Therefore, at this point, reducing the child’s behavioral problems through various intervention methods (special education, pharmacotherapy) will positively contribute to both the child’s and the caregiver’s stress and depression. In addition, psychosocial interventions related to the psychiatric problems of children with autism in public, which is the source of the stigmatization of the families of children with autism, may benefit autistic children and their caregivers. In light of the findings from various studies, there is a clear and pressing need for family-focused interventions that specifically target the internalized stigma experienced by caregivers of children with autism. The pervasive impact of stigma not only exacerbates the challenges faced by these caregivers but also has a ripple effect on the overall family dynamics and the well-being of the autistic child [53, 54]. Therefore, it is crucial to develop and implement interventions that address this stigma at its root. Such interventions should aim to educate and sensitize the broader community about autism, thereby reducing the instances of misguided perceptions and judgments [55]. Additionally, they should provide a supportive framework for families, offering them resources and strategies to cope with and counteract the effects of internalized stigma [53, 54].

These interventions could include psychoeducational programs for caregivers, focusing on enhancing their understanding of autism and its manifestations, and empowering them to navigate societal challenges with resilience and confidence [49, 56]. Support groups play a vital role here, offering a platform for sharing experiences and strategies, thus fostering a sense of community and collective strength [53, 54]. Furthermore, counseling and therapeutic services for caregivers should be integral to these interventions, addressing the psychological impact of caregiving and stigma, and promoting mental well-being [49].

Moreover, engaging in advocacy and awareness campaigns within communities can help change public attitudes, reducing the stigma associated with autism [55]. These campaigns could involve collaboration with schools, workplaces, and public spaces to create more inclusive and understanding environments [55, 57]. By implementing such comprehensive family-focused interventions, the adverse effects of internalized stigma can be significantly mitigated, improving the quality of life for both caregivers and children with autism [53, 54]. This approach not only benefits the individual families but also contributes to a more empathetic and informed society, paving the way for a future where autism is better understood and accepted [55, 57].

## Data Availability

The datasets generated during and/or analyzed during the current study are available from the corresponding author on reasonable request.
